# Does Distance Produce Beauty? The Influence of COVID-19 Lockdown on the Coach-Athlete Relationship in a Chinese Football School

**DOI:** 10.3389/fpsyg.2020.560638

**Published:** 2020-09-11

**Authors:** Juan Li, Hongyan Gao, Pan Liu, Caixia Zhong

**Affiliations:** ^1^School of Economics and Management, Beijing Jiaotong University, Beijing, China; ^2^Cardiff School of Sport and Health Sciences, Cardiff Metropolitan University, Cardiff, United Kingdom; ^3^Shandong Luneng Taishan Football School, Weifang, China

**Keywords:** COVID-19, lockdown, coach-athlete relationship, Chinese football school, improvement

## Abstract

This paper examined the relationship between coaches and youth athletes in China by comparing data collected before and after the lockdown. A total of 221 youth athletes aged 13–19 years in one professional football school completed coach-athlete relationship questionnaires. The rank-sum test was used to verify the differences in the data. The results of the Mann-Whitney U test showed that mean value of the three dimensions of the coach-athlete relationship (closeness, commitment, and complementarity) increased after the COVID-19 lockdown. The results also showed that athletes of different age categories showed different changes in the coach-athlete relationship after the lockdown, and the changes were not significantly related to the severity of the COVID-19 epidemic. The theoretical and practical implications are discussed.

## Introduction

COVID-19 is spreading rapidly around the world. To control the spread of the epidemic, countries with severe outbreaks, such as China, Italy, and the United Kingdom, have adopted different levels of lockdown measures. Among these, the lockdown measures adopted in China are probably the most stringent. The lockdown measures have exerted a severe impact on the economy and people’s lives. Taking China as an example, in the first quarter of 2020, GDP fell by 6.8% in comparison to the same period of the previous year (National Bureau of Statistics, 2020). In addition to affecting the economy, the lockdown policy also affects the people. Here, we are concerned with the impact of the lockdown on youth athletes in a Chinese professional football school. We are curious to determine whether the coach-athlete relationship has been influenced by the lockdown.

In recent years, researchers have addressed the relationship between coaches and athletes as a psychological phenomenon (Jowett and Arthur, 2019). This relationship is an essential concept in sports psychology and has received extensive attention (Horne and Carron, 1985; Philippe and Seiler, 2006; Yang and Jowett, 2016; Currie, 2019; Jowett and Arthur, 2019; Wachsmuth et al., 2020). The conceptual model of Wylleman (2000) asserted that the relationship between coaches and athletes could be defined based on the behavior of coaches and athletes on the playing field (Wylleman, 2000). LaVoi (2004) described the coach-athlete relationship in a sports environment as a sense of belonging and noted the possible personal benefits of closeness with others (LaVoi, 2004). Poczwardowski and colleagues (Poczwardowski, 1998; Poczwardowski et al., 2002) proposed the use of a qualitative research binary framework to study the coach-athlete relationship, conceptualizing it as a repeating pattern of mutual care between athletes and coaches (Poczwardowski et al., 2002). Jowett and her colleagues defined the coach-athlete relationship as “a social situation created from the interplay of their interpersonal feelings, thoughts, and behaviors” (Jowett, 2007; Jowett and Arthur, 2019, 430). In Jowett’s conceptual model, this relationship was characterized as comprising (a) closeness, which refers to the emotional bond established between coaches and athletes, reflected in their mutual trust and respect, emotional care and support, and interpersonal communication and appreciation; (b) commitment, which refers to the cognitive ties between coaches and athletes, which show that they are willing to maintain a close, long-term relationship; and (c) complementarity, which refers to the behavioral connection between coaches and athletes, expressed as a degree of collaboration and cooperation (Jowett and Arthur, 2019, 430).

The focus of each conceptual model above is different, but scholars seem to agree that the coach-athlete relationship is a mutual relationship between coaches and athletes (Wylleman, 2000; Poczwardowski et al., 2002; LaVoi, 2004; Jowett, 2007). Based on their research, we can propose an integrated coach-athlete relationship influence model: personal characteristics, social-cultural sports environment and relationship characteristics affect feelings (closeness), thoughts (commitment) and behavior (complementarity) through mutual communication, which once again influences intrapersonal outcomes, interpersonal outcomes and team outcomes (Jowett and Poczwardowski, 2007, 10). Communication is a bridge and bond between members of the coach-athlete relationship (Montgomery and Baxter, 1998; Jowett and Poczwardowski, 2007, 11). In other words, communication is the process of alienation, closeness, or even merging between coaches and athletes (Montgomery and Baxter, 1998; Jowett and Poczwardowski, 2007, 11). Communication is the basis for establishing a harmonious and stable coach-athlete relationship; in turn, the relationship will also affect communication (Montgomery and Baxter, 1998; Jowett and Poczwardowski, 2007, 11). Therefore, communication plays a very important role in the coach-athlete relationship. During the COVID-19 lockdown, the way people (including coaches and athletes) communicate has changed dramatically (Dalton et al., 2020; Yu et al., 2020). Will this extensive change in communication affect the coach-athlete relationship? This question deserves further study. Most existing empirical studies have focused on the antecedent variables (leadership, stress), outcome variables (burnout, satisfaction), and mediation effects (achievement goals) of coach-athlete relationships (Isoard-Gautheur et al., 2016; Thelwell et al., 2017; Davis et al., 2019). Some scholars have conducted comparative research based on coach-athlete relationships (Lenzen et al., 2004; Yang and Jowett, 2012). Existing comparative studies of coach-athlete relationships have focused on cross-cultural research (Yang and Jowett, 2012) and cross-sport types (Lenzen et al., 2004). To the best of our knowledge, no scholars have conducted comparative research on the coach-athlete relationship itself as related to the COVID-19 lockdown. To explore the impact of changes in communication on coach-athlete relationships, a comparative study of coach-athlete relationships data before and after the COVID-19 lockdown may be meaningful and innovative.

Sports psychologists have pointed out that new hypotheses related to theories are based on researchers’ observations related to social behavior (Jowett and Poczwardowski, 2007, 7). Such exploratory research will lead to an increase in our understanding of the theory (Jowett and Poczwardowski, 2007, 7). To better understand coach-athlete relationships, it is meaningful to continue to deepen the research on such relationships under the significant changes in communication caused by the COVID-19 lockdown.

## Materials and Methods

### Participants

With the consent of the football school and the participants’ legal guardian/next of kin, two hundred twenty-one male football players from the school agreed to complete the same questionnaire survey before the lockdown from January 4–17, 2020 and again after the lockdown on April 17, 2020. The mean age of the athletes was 15.11 years (*M*_*age*_ = 15.11 years, age range, 13–19 years, SD ± 1.82) before lockdown and 15.47 years (*M*_*age*_ = 15.47 years, age range, 13–19 years, SD ± 1.96) after lockdown. The sample distribution according to age is shown in Table 4. The regional distribution of participants was as follows: Anhui 6, Beijing 9, Fujian 2, Gansu 1, Guangdong 6, Guangxi 2, Hebei 4, Henan 8, Heilongjiang 1, Hunan 7, Jiangsu 5, Jiangxi 1, Liaoning 8, Ningxia 1, Shandong 113, Shnxi 1, Sichuan 5, Tianjin 3, Xinjiang 9, and Yunnan 5.

### Measures

We used the Chinese version (Yang and Jowett, 2010) of the Coach-Athlete Relationship Questionnaire (CART-Q), developed by Jowett and Ntoumanis (2004), in this study. The 11-item CART-Q was employed to assess athletes’ perceptions of the quality of the relationship with their coach. This questionnaire contained four items on closeness (e.g., I respect my coach), three items on commitment (e.g., I feel committed to my coach), and four items on complementarity (e.g., When I am coached by my coach, I feel at ease). A five-point Likert scale was used for the measures. The Cronbach alpha values of the questionnaire in this study were as follows: closeness 0.769, commitment 0.723, complementarity 0.668, and the whole scale 0.877 (before lockdown); closeness 0.763, commitment 0.754, complementarity 0.722, and the whole scale 0.902 (after lockdown). We conducted a confirmatory factor analysis of the scale, and the results showed that the scales had appropriate validity indicators (Eisinga et al., 2013). The results were as follows: KMO = 0.885, χ*^2^/df* = 1.842, GFI = 0.942, RMSEA = 0.067, RMR = 0.039, CFI = 0.968, NFI = 0.934, NNFI = 0.950 (before lockdown); KMO = 0.908, χ*^2^/df* = 1.767, GFI = 0.957, RMSEA = 0.062, RMR = 0.012, CFI = 0.983, NFI = 0.963, NNFI = 0.966 (after lockdown).

### Procedure

This study was carried out in accordance with the recommendations and ethical guidelines of the Ethical Review Board of Beijing Jiaotong University JG201905017. The protocol was approved by the Ethical Review Board of Beijing Jiaotong University. All subjects gave written informed consent in accordance with the Declaration of Helsinki. This study was conducted at a Chinese professional football school that was closed for 3 months under the COVID-19 lockdown. During these participants’ time at the school before the COVID-19 lockdown, coaches were responsible for the cultivation of football skills among the youth players. To improve training efficiency and effectiveness, coaches usually lived in campus dorms. The sudden outbreak of COVID-19 forced the players to return home and stay at home for a long time due to the lockdown. The participants ended their winter training and returned to their homes around January 20. At that time, online teaching of general knowledge by teachers and online professional training by coaches were adopted, using WeChat, WeChat groups, and telephone instant messaging to manage the learning and training of athletes.

The data were collected twice, and the two questionnaires had the same content. The first data collection procedure used a paper questionnaire, and the participating athletes completed the questionnaire in the classroom. During the completion process, the coach and teacher were not present. The researchers distributed the questionnaire to the athletes and explained to them that the purpose of the questionnaire survey was scientific research and that it needed to be completed anonymously and independently according to their own understanding. The athletes were given 5 min to fill out the questionnaires, and then the researchers collected them all. The entire survey, including the instructions, took a total of 10 min. Due to the limitations of the research conditions, it was not possible for all athletes to complete the questionnaires at the same time, so the first group of questionnaires were completed and collected within a 2-week period. After the questionnaires were collected, they were manually encoded and entered into the computer, thereby constituting the first data set of this study.

Casler et al. (2013) has noted that electronic questionnaires can obtain data characteristics similar to paper questionnaires. The athletes were distributed in their homes in 20 provinces after they left Shandong Luneng Taishan Football School. They had not yet returned to the school, and it was thus impossible to collect the paper questionnaire again. Therefore, the second survey took the form of an electronic questionnaire. During the completion process, neither the coach nor the teacher was present. The researchers sent the electronic questionnaire to the athletes through the WeChat group and explained to them that the purpose of the questionnaire survey was scientific research and that the survey needed to be filled out anonymously and independently according to their own understanding. The athletes completed the response and submission of the questionnaire on April 17. We obtained the secondary data of this study from the questionnaire system on April 18.

We cleaned the survey data by deleting scales with open items, all answers the same, contradictory answers or more than one answer. The electronic questionnaire was tested before it was issued. The test showed that the questionnaire response time was 40–50 s. Given the youths’ cognitive level, we deleted any scales with a response time within 60 s (answering too quickly may reflect a coping attitude and may pollute the data). Therefore, we obtained 190 (86%) valid questionnaires for the first survey and 197 (89%) for the second survey. The respondents were not compensated for their participation in the study. The researchers informed the participants about the purpose of the study (for research only) and that the questionnaires were anonymous. In addition, respondents were told to complete the scales alone.

### Statistical Analysis

We used SPSS software version 25.0, The The SPSSAU project (2020), and Microsoft Excel software to analyze the sample data obtained through measuring the three dimensions of the coach-athlete relationship before and after the COVID-19 lockdown. Distribution normality was assessed using the Kolmogorov–Smirnov test. Normally distributed data were analyzed with the T-test, while non-normally distributed data were analyzed with the rank-sum test. We tested the significance level (Table 1) and Pearson correlation coefficient of each test variable and conducted comparative studies based on age groupings and regional differences.

**TABLE 1:** Significance of the variables.

	**Dimensions**	**α**	**α**	**KMO**	**χ ^2^/*df***	**GFI**	**RMSEA**	**RMR**	**CFI**	**NFI**	**NNFI**
1	Closeness	0.769	0.877	0.885	1.842	0.942	0.067	0.039	0.968	0.934	0.950
	Commitment	0.723									
	Complementarity	0.668									
2	Closeness	0.763	0.902	0.908	1.767	0.957	0.062	0.012	0.983	0.963	0.966
	Commitment	0.754									
	Complementarity	0.722									

*1 = sample before COVID-19 lockdown; 2 = sample after COVID-19 lockdown; α = Cronbach α; KMO = KMO and Bartlett test.*

## Results

### Normality Test

In this study, the Kolmogorov–Smirnov test was used to evaluate distribution normality. As shown in Table 2, the test results showed that the samples collected before and after the COVID-19 lockdown did not conform to a normal distribution, and thus the rank-sum test should be used to verify the differences in the data.

**TABLE 2:** Normality test of data before and after lockdown.

	**Variables**	***N***	**Normality**	**Extreme difference**			**Kolmogorov–Smirnov Z**	***Sig.***
		
			**Mean ± SD**	**Absolute**	**Plus**	**Minus**		
1	Closeness	190	4.514 ± 0.610	0.229	0.213	−0.229	0.229	0.000
	Commitment	190	4.072 ± 0.787	0.154	0.119	−0.154	0.154	0.000
	Complementarity	190	4.270 ± 0.637	0.148	0.126	−0.148	0.148	0.000
2	Closeness	197	4.799 ± 0.393	0.381	0.305	−0.381	0.381	0.000
	Commitment	197	4.592 ± 0.575	0.284	0.239	−0.284	0.284	0.000
	Complementarity	197	4.638 ± 0.496	0.244	0.233	−0.244	0.244	0.000

*N, number of samples; 1 = sample before COVID-19 lockdown; 2 = sample after COVID-19 lockdown.*

### Average Value Difference Examination

#### Difference Examination

Because the samples did not conform to a normal distribution, the rank-sum test was used to verify the differences in the data. The Mann-Whitney U significance test method was thus adopted to verify the differences in the samples collected before and after lockdown. As Table 3 shows, the results showed that athletes’ closeness to coaches before COVID-19 lockdown was significantly different from that after the COVID-19 lockdown (*Z* = −5.390, *p* < 0.001), as was their commitment to coaches (*Z* = −7.586, *p* < 0.001) and their complementarity with coaches (*Z* = −6.068, *p* < 0.001).

**TABLE 3:** Testing of significant differences and comparison of samples.

**Variables**	**Group**	***N***	**Mean ± SD**	**Mean rank**	**Sum of ranks**	**Mann-Whitney U**	**Wilcoxon W**	***Z***	***Sig.***
Closeness	1	190	4.514 ± 0.610	165.82	31506.50	13361.500	31506.500	−5.390	0.000
	2	197	4.799 ± 0.393	221.18	43571.50				
Commitment	1	190	4.072 ± 0.787	151.28	28742.50	10597.500	28742.500	−7.586	0.000
	2	197	4.592 ± 0.575	235.21	46335.50				
Complementarity	1	190	4.270 ± 0.637	159.92	30385.00	12240.000	30385.000	−6.068	0.000
	2	197	4.638 ± 0.496	226.87	44693.00				

*N, number of samples; 1 = sample before COVID-19 lockdown; 2 = sample after COVID-19 lockdown.*

Next, we compared the differences between the two groups. As Table 3 shows, the mean value of closeness after the COVID-19 lockdown was higher than that before (4.799 > 4.514), the mean value of commitment after the COVID-19 lockdown was higher than that before (4.592 > 4.072), and the mean value of complementarity after the COVID-19 lockdown was also higher than that before (4.638 > 4.270).

#### Analysis by Age

We next compared and analyzed the data of the three dimensions of coach-athlete relationship before and after the COVID-19 lockdown according to age (see Table 4). As shown in the table, in the 13-, 14-, 15-, 16-, 17-, and 19-year-old age groups, the values of closeness, commitment, and complementarity significantly increased after lockdown. We separately measured the percentage change in the mean according to age by group in the following order: 13-, 14-, 15-, 16-, 17-, and 19-year-olds. The increases in closeness by age group were 1.97%, 3.49%, 10.90%, 7.40%, 9.13%, and 15.12%, respectively. Among these groups, the increase is largest for the 19-year-old group and is the smallest for the 13-year-old group. Similarly, the increases in age in terms of commitment were, respectively, 12.79%, 7.13%, 19.3%, 13.96%, 10.30%, and 26.23%. Among these groups, the data show the greatest increase for the 19-year-olds; however, unlike the closeness index, the data for commitment is the smallest for the 14-year-olds. Finally, in terms of complementarity, each age group also improved as follows: 3.32%, 5.25%, 20.64%, 7.24%, 11.05%, and 12.30%, respectively. Unlike the other two variables, the data for complementarity show the greatest improvement for the 15-year-olds and the smallest improvement for the 13-year-olds.

**TABLE 4:** Analysis results based on age.

	**Age**	**N**	**Closeness**		**Commitment**		**Complementarity**	
			**Mean ± SD**	**Increase**	**Mean ± SD**	**Increase**	**Mean ± SD**	**Increase**
1	13	39	4.76 ± 0.60	1.89%	4.20 ± 1.11	12.79%	4.51 ± 0.88	3.32%
2		45	4.85 ± 0.41		4.73 ± 0.52		4.66 ± 0.68	
1	14	49	4.76 ± 0.55	3.49%	4.42 ± 0.74	7.13%	4.53 ± 0.82	5.25%
2		19	4.92 ± 0.27		4.74 ± 0.51		4.76 ± 0.56	
1	15	35	4.30 ± 1.03	10.90%	3.80 ± 1.09	19.30%	3.83 ± 1.28	20.64%
2		40	4.77 ± 0.49		4.53 ± 0.76		4.62 ± 0.66	
1	16	24	4.48 ± 0.76	7.40%	4.04 ± 0.95	13.96%	4.30 ± 0.89	7.24%
2		33	4.81 ± 0.57		4.61 ± 0.75		4.61 ± 0.78	
1	17	24	4.21 ± 0.87	9.13%	3.81 ± 0.84	10.30%	3.97 ± 0.98	11.05%
2		27	4.59 ± 0.75		4.20 ± 0.97		4.41 ± 0.89	
1	18							
2		13	4.79 ± 0.45		4.49 ± 0.75		4.67 ± 0.61	
1	19	19	4.22 ± 0.91	15.12%	3.79 ± 1.21	26.23%	4.26 ± 0.77	12.30%
2		20	4.86 ± 0.47		4.78 ± 0.52		4.79 ± 0.54	

*N, number of samples; 1 = sample before COVID-19 lockdown; 2 = sample after COVID-19 lockdown.*

#### Analysis by Region

**FIGURE 1:**
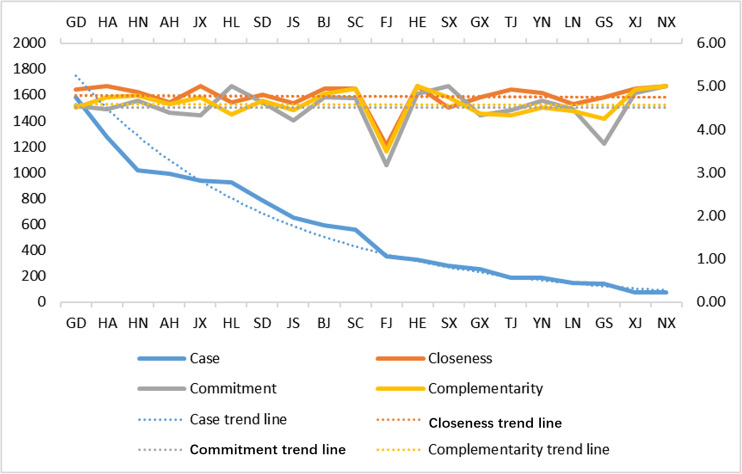
Comparison of regional confirmed cases and CAR mean level. Case: number of COVID-19 confirmed cases by region; Data source: China National Health Commission, provincial and municipal health committees, provincial and municipal governments, Hong Kong, Macao and Taiwan official channels. Data as of April 25, 2020 1:51. English abbreviations of Chinese provinces: Ningxia NX, Xinjiang XJ, Gansu GS, Liaoning LN, Yunnan YN, Tianjin TJ, Guangxi GX, Shanxi SX, Hebei HE, Fujian FJ, Sichuan SC, Beijing BJ, Jiangsu JS, Shandong SD, Heilongjiang HL, Jiangxi JX, Anhui AN, Hunan HN, Henan HA, Guangdong GD.

We used correlation analysis to study the correlations among regional confirmed COVID-19 cases and closeness, commitment, and complementarity, using the Pearson correlation coefficient to indicate the strength of the correlation (Arndt et al., 1999; Hauke and Kossowski, 2011). Specific analysis showed the following (see Table 5): confirmed cases and closeness, commitment, and complementarity did not show significant correlations among the three items; the correlation coefficient values were 0.155, 0.056, 0.074, respectively; all were close to 0; and the *p* values were all greater than 0.05, indicating that there were no significant correlations among confirmed cases and closeness, commitment, and complementarity. Figure 1 represents the actual relationships among these variables. The trend line of the number of confirmed cases in various regions has no common or opposite trend with the coach-athlete relationship level before or after lockdown, as shown in Figure 1.

**TABLE 5:** Correlation analysis results of CAR and regional confirmed cases.

	**Mean ± SD**	**Case**	**Closeness**	**Commitment**	**Complementarity**
Case	567.85 ± 438.982	1			
Closeness	4.763 ± 0.311	0.155	1		
Commitment	4.506 ± 0.447	0.056	0.653**	1	
Complementarity	4.583 ± 0.347	0.074	0.820**	0.805**	1

**p < 0.01; Case: COVID-19 Regional Confirmed Cases.

## Discussion

The main purpose of this study was to examine the changes in the coach-athlete relationship from before to after lockdown. The research provided important information for the academic community on the cultivation of youth athletes. The establishment and application of multiple communication methods, combined with tension-relief and relaxation training methods, might help to better develop coach-athlete relationships.

The first conclusion we draw is that the mean values of the three dimensions of coach-athlete relationship after lockdown are higher than those before lockdown, which is contrary to our expected result based on common sense (Przybylski and Weinstein, 2013). We expected the coach-athlete relationship to be negatively affected due to lockdown. The second conclusion is that lockdown has different effects on closeness, commitment, and complementarity at different ages. This point is a more in-depth analysis of the first conclusion. The comparison results for the various age groups are different, which is consistent with the trend of the first conclusion. The third conclusion is that the relationship between the impact of lockdown on the coach-athlete relationship and the severity of the epidemic of each province is not significant. Through this study, we evaluated each of the dimensions of the CART-Q, which would be very useful for future researchers to better understand what this instrument evaluates. The following factors may be involved in these findings. First, lockdown changes the way coaches and athletes communicate (Dalton et al., 2020; Yu et al., 2020). Indirect online communication reduces the sense of leadership (coach) status and shortens the psychological distance between leaders (coach) and their subordinates (athletes). This kind of communication avoids the reduced communication effect caused by the unequal status (Remland, 1984) and psychological distance (Brunelle, 2013) experienced in face-to-face communication between the superior and the subordinate. These changes in communication methods and the long-term lockdown may make athletes miss their training life with their coaches and teammates and, consequently, make them more eager to return to the training ground, thereby improving the relationship between coaches and athletes from the perspective of athletes. These postulations may require deeper empirical research to prove. In addition to these possible causes, we would assert that there are other reasons for this change.

The face-to-face communication relationship is more inclined toward hierarchical and one-way communication (Sannomiya and Kawaguchi, 1999; Kirkman et al., 2004); after the lockdown, a greater use of social media created more equal and diversified communication (Clarke and Eslami, 2019). However, if no previous foundation has been built through face-to-face communication, it is impossible to engage in truly effective social media communication (Dunbar, 2016). Lockdown gives more opportunities to think, supplement and correct existing interpersonal relationships (Pan et al., 2020). These two communication methods are complementary (Casler et al., 2013; Dunbar, 2016). Therefore, we should give full play to the role of multiple communication media to encourage athletes to actively participate in decision-making and learn from one another (Wu et al., 2016). In addition, lockdown also allows for more empathy, mutual care and concern between the two sides of the exchange (Pan et al., 2020), which lays the foundation for establishing a good relationship after returning to work and school (Westerman et al., 2016).

Second, the lockdown has changed the communication environment between coaches and athletes. Athletes accept militarized boarding management in football schools, and families play a limited direct role in communication. Although coaches and athletes communicate face to face, players lack family support and security. When athletes are at home and participate in online communication, the family gives the athletes support and a sense of security (Fitriana and Xin, 2019). The athletes may then dare to communicate with the coach with a more equal and peaceful attitude and accept the coach’s guidance. From this hypothesis, we also infer that the family’s role for athletes during lockdown is not only communicating but also providing a warm and harmonious environment (Fitriana and Xin, 2019). Athletes returning home on vacation experience a kind of psychological relaxation and adjustment (Kühnel and Sonnentag, 2011). Proper vacations are conducive to the growth of athletes and help reduce the effects of burnout (Lemyre et al., 2007). This situation also proves that family support can be conducive to athletes’ progress, better coach-athlete relationships and superior performance (Fitriana and Xin, 2019).

These results lead us to think deeply. Should young athletes differentiate their mental training content by age in Chinese football school? The difference between the mean before and after lockdown according to age provides us with certain empirical evidence. Moreover, will the diligent and controlled method advocated by football schools lead to psychological disorders in young athletes? In the past, we have advocated not wasting time, orienting training toward efficiency, and ignoring the role of relaxation in life and training, which is, in fact, not conducive to the recovery of youth athletes’ mental health (Li, 2019). The goal of coaches to improve performance-oriented training ignores athletes’ other psychological needs, resulting in the failure of the coach-athlete relationship to attain a high level (Li, 2019). Shandong Luneng Taishan Football School delivered more than 230 players to the Chinese national football team at all levels (Chinese Football Association, 2019). The data survey based on this case reflects some issues in talent training and provides a reference for future training models.

As discussed above, the lockdown has changed the method and content of communication between coaches and athletes. From the results of geographic analysis, it can be seen that the average value of coach-athlete relationship has no significant correlation with the number of confirmed cases in the region. The above content has analyzed the reasons for the change of the mean, which further shows that online education is not easily restricted by social emergencies, and also shows the advantages of online education (Xinhuanet, 2020). Further, we have found from observation and investigation that during face-to-face training between athletes and coaches, the coaches offered assistance through on-site teaching tools for on-site professional training, personal demonstrations, and demonstration by high-performing team members. In online teaching, to express the training content more vividly, the coaches use more words to describe the training content, which muscles should be trained to obtain specific types of strength, and which muscle groups are being exercised in each training drill (evidence found through observation; Xu, 2017). In front of the camera, the coach used more body language to demonstrate the teaching content (Yue and Zou, 2019). For more effective expression, the coach used more vibrant language in descriptions (Xu, 2017; Yue and Zou, 2019). In addition, the coach was able to observe the movement of each athlete through the camera and make timely corrections (Xu, 2017; Yue and Zou, 2019). These changes are positive and may be effective in improving coach-athlete relationship.

This research offers many inspiring ideas for practical application: (a) We will usher in the explosion of online education and diversification of online teaching methods (Weisband and Atwater, 1999; Radha et al., 2020; Su, 2020). Online teaching has been carried out for years, but there has been resistance to placing great pressure on a single group and taking a full 3-month period to conduct online teaching to verify the effect of this approach. Lockdown has provided us with an opportunity to verify the effectiveness of online teaching (Shenoy et al., 2020; Su, 2020). Therefore, it is expected that the lockdown will usher in the explosive development of online teaching for years into the future (Yue and Zou, 2019; Radha et al., 2020; Shenoy et al., 2020; Su, 2020). This situation is an opportunity for online educators and a challenge to traditional tutoring and training institutions (Shenoy et al., 2020; Su, 2020). It may be that some offline education and training institutions will face challenges to their survival (Su, 2020).

(b) We can improve employment and change people’s perception of new occupations. At present, people’s perception of online anchors has a specific entertainment value (He, 2019). During the lockdown period, Chinese teachers have become online anchors, and they have also produced many internet humorous videos for various reasons (Zhang, 2020). These internet jokes include the following: *the most tired anchor of the live-broadcast classroom: the physical education teacher; the online classroom in the epidemic situation: the embarrassing “teacher anchor”; and the teacher is also crazy: I broadcast the online class at home, and I crashed faster than the live-broadcast software* (Tencent, 2020). Coaches at football schools have also had to lead players to train at home through live webcasts. The findings of this study confirm that live-broadcast courses that can effectively control the classroom are practical (Yue and Zou, 2019; Zhang, 2020). This finding may develop another career: online sports coaching, which is not restricted by space, geography, or time (coaches give physical education lessons through online media). There is potentially a variety of teaching methods and a variety of class sizes. Live broadcasts to homes will allow students to be recruited from all over the world and will enable the full sharing of global resources (Yue and Zou, 2019).

(c) We will save social resources. Most sports projects have venue requirements (Yue and Zou, 2019). If football training can be combined with offline training in the future, in places where the venue resources are insufficient, it will improve the efficiency of venue utilization, reduce the cost of sports training, and reduce the space required for sports grounds (Yue and Zou, 2019). This study also has limitations. First, when we first collected data, we did not predict that the lockdown would occur, so the first dataset did not include geographic information. This omission made it impossible for us to conduct a comparative analysis by region before and after lockdown, and we cannot obtain a more in-depth analytical result. Furthermore, this study is limited by the source and number of samples. The regional sample size distribution is uneven, and the interpretation of the results may produce biased conclusions. Finally, this study selected one school as the research object, and the sample size is limited. Due to the different requirements of the education authorities in different regions, the specific implementation measures of the school are unique to that region. Due to the limitations of the school’s supporting teaching methods, the situation of each school may vary.

## Conclusion

The multimedia teaching method of communication adopted during lockdown gave us an opportunity to reexamine the coach-athlete relationship. The results of the study showed that during the lockdown period, remote communication methods such as multimedia based on face-to-face communication, assisted by a loose communication environment and rich communication content, promoted the improved quality of the coach-athlete relationship as experienced by athletes.

The coach-athlete relationship is an essential variable in sports teams (Côté and Gilbert, 2009; Yang and Jowett, 2012). Its improvement will bring a chain reaction, fostering satisfaction (Lorimer and Jowett, 2009), reduced burnout (Isoard-Gautheur et al., 2016), organizational citizenship behavior (Cummins and Spencer, 2015), performance (Jowett, 2017), team cohesion (Jowett and Chaundy, 2004) and other changes. To assess the degree of impact on the other variables due to the lockdown, further research is needed for observation and investigation.

Several Chinese leaders have mentioned that football should start with children (Hanwang-Wuhan Evening News, 2014), and the professional ability training of young athletes relies on a good relationship between coaches and athletes (Jiang, 2018). This goal requires us to fully understand the coach-athlete relationship. In the field of Chinese youth football training, such understanding requires more in-depth scientific research.

## Data Availability Statement

The raw data supporting the conclusions of this article will be made available by the authors, without undue reservation.

## Ethics Statement

The studies involving human participants were reviewed and approved by Beijing Jiaotong University. The participants, and where necessary, the participants’ legal guardian/next of kin provided written informed consent to participate in this study.

## Author Contributions

JL designed the research and completed the manuscript. HG designed the research with JL and proposed the discussion. CZ completed the data collection. PL completed most of the data analysis and writing of the results. All authors contributed to the article and approved the submitted version.

## Conflict of Interest

The authors declare that the research was conducted in the absence of any commercial or financial relationships that could be construed as a potential conflict of interest.
